# Supplementary material for: Nucleophilic Radiofluorination Using Tri-tert-Butanol Ammonium as a Bifunctional Organocatalyst: Mechanism and Energetics
Source: Molecules. 2022 Feb 3;27(3):1044. doi: 10.3390/molecules27031044 (PMC8838713; doi:10.3390/molecules27031044)
Supplement: Supplementary file 1 [file molecules-27-01044-s001.zip › molecules-1553563-supplementary.pdf]

molecules

Supplementary Materials

Article

Nucleophilic Radiofluorination Using Tri-*tert*-Butanol Ammonium as a Bifunctional Organocatalyst: Mechanism and Energetics

Young-Ho Oh [1], Sandip S. Shinde [2,*] and Sungyul Lee [1,*]

[1] Department of Applied Chemistry, Kyung Hee University, Deogyeong-daero 1732, Yongin-si 17104, Gyeonggi-do, Korea; chem_yhoh@daum.net

[2] Department of Nuclear Medicine, Molecular Imaging and Radiochemistry, Friedrich-Alexander University Erlangen-Nürnberg (FAU), 91054 Erlangen, Germany

* Correspondence: shinde88@gmail.com (S.S.S.); sylee@khu.ac.kr (S.L.)

Structures and Cartesian Coordinates

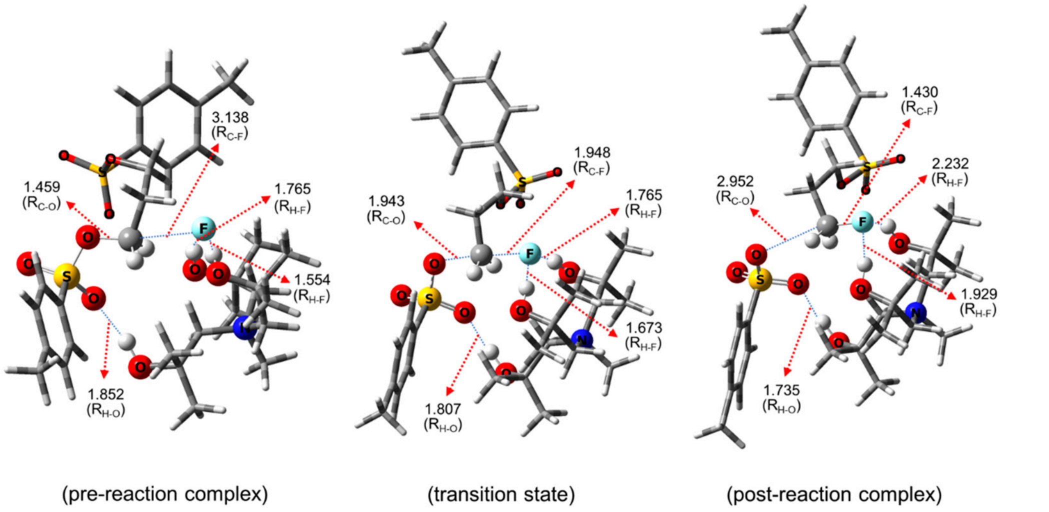

Figure S1. Pre– and post–reaction complexes and the transition states of [^{18}F]–Fluorination using TBMA–^{18}F in CH_3CN.

SN2_1: pre-reaction complex
C 0.8347910000 -1.3320370000 1.0876210000
H 1.7835920000 -0.9180830000 1.4391210000
H 0.2920140000 -0.6007470000 0.4885740000
C -0.0092780000 -1.8079700000 2.2497010000
H 0.4380510000 -2.6967240000 2.7020720000
C -1.4638080000 -2.0464460000 1.9030320000
O 1.1250810000 -2.4940690000 0.2539520000
S 1.9803150000 -2.2489410000 -1.0612740000
O 1.6275980000 -0.9700380000 -1.6555720000
O 1.8300580000 -3.4542360000 -1.8425670000
C 3.6342830000 -2.1350480000 -0.4388000000
C 4.4538100000 -1.0984180000 -0.8609660000
C 4.0970840000 -3.1336720000 0.4178520000
C 5.7682450000 -1.0584720000 -0.4046560000
H 4.0772110000 -0.3268510000 -1.5223350000
C 5.4066940000 -3.0725200000 0.8627060000
H 3.4421470000 -3.9366660000 0.7366700000
C 6.2606460000 -2.0378890000 0.4567890000
H 6.4175130000 -0.2513850000 -0.7255570000
H 5.7793210000 -3.8385160000 1.5342580000
C 7.6781530000 -1.9950870000 0.9550700000
H 8.2267990000 -1.1631500000 0.5132300000
H 7.6970950000 -1.8850480000 2.0424340000
H 8.1980700000 -2.9249180000 0.7120310000
O -1.6112190000 -3.1946310000 1.0159560000
S -2.1467810000 -2.9364150000 -0.4668790000
O -2.2482860000 -4.2633150000 -1.0358820000
O -1.3421140000 -1.9334930000 -1.1280010000
C -3.7645270000 -2.2665540000 -0.1939800000
C -4.7158970000 -3.0585130000 0.4472720000
C -4.0522190000 -0.9764800000 -0.6181680000
C -5.9809320000 -2.5370560000 0.6609650000

H -4.4704200000 -4.0634380000 0.7720440000
C -5.3321840000 -0.4748060000 -0.3978550000
H -3.2959910000 -0.3768910000 -1.1133670000
C -6.3070410000 -1.2405980000 0.2410760000
H -6.7316590000 -3.1405390000 1.1599150000
H -5.5730780000 0.5302400000 -0.7262780000
C -7.6893850000 -0.6992730000 0.4781660000
H -8.4298310000 -1.2916000000 -0.0658030000
H -7.9455060000 -0.7514350000 1.5391010000
H -7.7681530000 0.3375390000 0.1507640000
F -1.4916380000 0.7662940000 0.9019880000
H -2.0394820000 -2.2994880000 2.7933100000
H -1.8710560000 -1.1474280000 1.4354890000
N 0.1498090000 3.8141200000 -0.1931030000
C 0.6884120000 4.0763670000 1.2062300000
C 0.4565030000 3.0555780000 2.3647030000
H 0.2542620000 5.0336490000 1.5027560000
H 1.7572950000 4.2291710000 1.0610420000
O 0.6232980000 1.7143280000 1.9602690000
C -0.8813450000 3.2513900000 3.0961160000
H -0.8548430000 2.6628800000 4.0163410000
H -1.7459740000 2.9184400000 2.5243890000
H -1.0252240000 4.3007490000 3.3649160000
C 1.5666940000 3.3633040000 3.3748020000
H 2.5508190000 3.1746310000 2.9431350000
H 1.4408060000 2.7187470000 4.2465190000
H 1.5217780000 4.4056190000 3.7012410000
H -0.2270480000 1.3483180000 1.5920160000
C 0.8294910000 2.6635300000 -0.9197480000
C 2.3713160000 2.5581080000 -1.0981340000
C -1.3362270000 3.5197190000 -0.0729130000
C -2.1666270000 2.9701880000 -1.2563960000
C -2.1012050000 3.7535800000 -2.5614200000
C -3.6096000000 2.9839420000 -0.7277520000
O -1.8044200000 1.6304740000 -1.5505310000
C 3.0829790000 3.8203530000 -1.5644000000
O 2.4958760000 1.6496200000 -2.1978540000
C 3.0886190000 1.9496900000 0.1023170000
H 0.4131640000 2.6945850000 -1.9225620000
H 0.4823290000 1.7529120000 -0.4350560000
H 2.5700820000 4.2766870000 -2.4132240000
H 4.0862540000 3.5314430000 -1.8847600000
H 3.1828560000 4.5524170000 -0.7613500000
H 3.0745360000 2.6016300000 0.9738690000
H 4.1301100000 1.7684670000 -0.1754610000
H 2.6208340000 1.0037960000 0.3853050000
H 2.1398130000 0.7874000000 -1.9360260000
H -1.7789930000 4.4444180000 0.2992860000
H -1.4161900000 2.7478880000 0.6794070000
H -1.7060330000 1.1611280000 -0.6877870000
H -2.2881390000 4.8168050000 -2.3941540000
H -2.8696760000 3.3679540000 -3.2354920000
H -1.1382630000 3.6330790000 -3.0594110000
H -3.9656430000 3.9992630000 -0.5360920000

H -3.6776690000 2.4017530000 0.1957890000
H -4.2544160000 2.5197120000 -1.4768930000
C 0.3332250000 5.1007110000 -0.9478790000
H 1.3239690000 5.4954810000 -0.7498590000
H -0.4142800000 5.8080340000 -0.5941210000
H 0.2101080000 4.9185950000 -2.0111370000
H 0.0052890000 -1.0111280000 2.9996970000

Electronic Energy (Hartree)	Zero Point Correction (kcal/mol)	Electronic and Zero Point Correction (Hartree)	Gibbs Free Energy (kcal/mol)	Electronic and Gibbs Free Energy (Hartree)	Number of Imaginary Frequencies
−2800.926961	498.2099125	−2800.133	428.3135118	−2800.2444	0

SN2_1: transition state
C 0.3796940000 0.6112400000 0.9700730000
H -0.5670560000 0.8747770000 1.4141080000
H 0.3919130000 0.2646390000 -0.0499210000
C 1.6329420000 0.6273570000 1.7966940000
H 2.0598080000 1.6295650000 1.7677220000
C 2.6596680000 -0.3789260000 1.3243190000
O 0.5501520000 2.4320200000 0.3139450000
S -0.2611330000 2.8180020000 -0.9005700000
O -0.5841890000 1.6238760000 -1.7047360000
O 0.3687820000 3.9037140000 -1.6431230000
C -1.8162660000 3.4105170000 -0.2684110000
C -2.7858650000 3.8050830000 -1.1898580000
C -2.0729700000 3.4314000000 1.0943290000
C -4.0233820000 4.2235440000 -0.7287560000
H -2.5768340000 3.7801590000 -2.2538080000
C -3.3272290000 3.8436270000 1.5405270000
H -1.3067820000 3.1300200000 1.7985870000
C -4.3156120000 4.2409920000 0.6413900000
H -4.7834430000 4.5325250000 -1.4389990000
H -3.5362550000 3.8572010000 2.6047370000
C -5.6757470000 4.6736470000 1.1164000000
H -5.9216290000 5.6663390000 0.7317160000
H -6.4436090000 3.9842560000 0.7548930000
H -5.7234120000 4.7009890000 2.2053840000
O 2.8736810000 -0.1323770000 -0.0908760000
S 4.0534500000 -0.9449790000 -0.8064870000
O 3.8673100000 -0.6830830000 -2.2142380000
O 4.0630570000 -2.3068380000 -0.3147730000
C 5.5014520000 -0.1124060000 -0.2257730000
C 5.7252980000 1.1995400000 -0.6417230000
C 6.3717770000 -0.7653650000 0.6353080000
C 6.8524830000 1.8577960000 -0.1808200000
H 5.0333610000 1.6919880000 -1.3154140000
C 7.5007080000 -0.0862400000 1.0838060000
H 6.1744710000 -1.7847160000 0.9437150000
C 7.7549400000 1.2259280000 0.6857560000
H 7.0420170000 2.8781750000 -0.4961980000
H 8.1917650000 -0.5859210000 1.7533190000
C 8.9718860000 1.9639070000 1.1691470000
H 9.5832850000 2.2881070000 0.3233740000

H 8.6801800000 2.8598950000 1.7229620000
H 9.5821710000 1.3369780000 1.8192840000
F -0.0362660000 -1.2293750000 1.4539610000
H 3.6009690000 -0.2399910000 1.8625090000
H 2.3016140000 -1.3988350000 1.4448580000
N -2.9431400000 -2.7661220000 -0.3183600000
C -3.8794730000 -2.5956500000 0.8707350000
C -3.3913170000 -1.9384440000 2.1960220000
H -4.2325180000 -3.6028510000 1.1009810000
H -4.7231800000 -2.0326590000 0.4751800000
O -2.5866800000 -0.7957030000 1.9750670000
C -2.7059820000 -2.9181040000 3.1593010000
H -2.5957490000 -2.4237000000 4.1270940000
H -1.7136000000 -3.2318760000 2.8375390000
H -3.3198780000 -3.8111150000 3.2984550000
C -4.6705910000 -1.4495070000 2.8804620000
H -5.1656050000 -0.6854560000 2.2789690000
H -4.4126210000 -1.0176290000 3.8488470000
H -5.3677460000 -2.2760030000 3.0396100000
H -1.6473630000 -1.0483110000 1.8672210000
C -2.5550680000 -1.4647870000 -1.0096640000
C -3.6037020000 -0.4249220000 -1.5022780000
C -1.6871200000 -3.4586100000 0.1761260000
C -0.4518260000 -3.6704610000 -0.7281420000
C -0.6894110000 -4.3136310000 -2.0877170000
C 0.4657170000 -4.5748750000 0.1074460000
O 0.2114010000 -2.4365170000 -0.9670670000
C -4.8040810000 -0.9888710000 -2.2516460000
O -2.8881110000 0.3156410000 -2.4885900000
C -4.0623600000 0.5383210000 -0.4114530000
H -2.0015740000 -1.7788010000 -1.8907910000
H -1.8622480000 -0.9600490000 -0.3376680000
H -4.4941380000 -1.6908110000 -3.0279380000
H -5.3083490000 -0.1485210000 -2.7333950000
H -5.5189460000 -1.4734520000 -1.5850420000
H -4.6760740000 0.0541740000 0.3467090000
H -4.6514490000 1.3283770000 -0.8830250000
H -3.2014190000 0.9846300000 0.0909500000
H -2.1385720000 0.7827250000 -2.0807870000
H -2.0279640000 -4.4165540000 0.5719640000
H -1.3214740000 -2.8476880000 0.9916760000
H 0.2932070000 -1.9838880000 -0.1072560000
H -1.2849850000 -5.2241670000 -1.9966380000
H 0.2805700000 -4.5747060000 -2.5169450000
H -1.1812500000 -3.6284770000 -2.7789620000
H 0.0402060000 -5.5732370000 0.2333300000
H 0.6387460000 -4.1391490000 1.0961990000
H 1.4257250000 -4.6605970000 -0.4045200000
C -3.6688460000 -3.6785180000 -1.2706450000
H -4.7127320000 -3.3869740000 -1.3196390000
H -3.5958850000 -4.6943230000 -0.8872190000
H -3.2186460000 -3.6087820000 -2.2551720000
H 1.3827710000 0.3952120000 2.8326260000

Electronic Energy (Hartree)	Zero point Correction (kcal/mol)	Electronic and Zero Point Correction (Hartree)	Gibbs Free Energy (kcal/mol)	Electronic and Gibbs Free Energy (Hartree)	Number of Imaginary Frequencies
−2800.888529	497.6915897	−2800.0954	427.5297525	−2800.207217	1

SN2_1: post-reaction complex
C 0.4454830000 0.1355960000 1.6923170000
H -0.4046260000 0.5134620000 2.2558760000
H 0.2647890000 0.2684170000 0.6259880000
C 1.7453540000 0.7485300000 2.1485990000
H 1.7145090000 1.8110220000 1.8989770000
C 2.9511980000 0.0859810000 1.5192200000
O -0.6167880000 2.8723490000 1.3830970000
S -1.0409200000 2.9907690000 -0.0215780000
O -0.8993090000 1.7076090000 -0.7626870000
O -0.4362220000 4.1217330000 -0.7411940000
C -2.8048100000 3.2982190000 0.0049740000
C -3.4672000000 3.5036290000 -1.2042810000
C -3.5076440000 3.2769050000 1.1997810000
C -4.8409170000 3.6935850000 -1.2055100000
H -2.9120580000 3.5139200000 -2.1356480000
C -4.8890160000 3.4642900000 1.1853520000
H -2.9772290000 3.1098420000 2.1296860000
C -5.5729820000 3.6748780000 -0.0112370000
H -5.3600370000 3.8549140000 -2.1450680000
H -5.4414920000 3.4462900000 2.1189650000
C -7.0628070000 3.8884640000 -0.0330210000
H -7.3007630000 4.9053550000 -0.3570540000
H -7.5431690000 3.2022060000 -0.7345520000
H -7.4973750000 3.7348770000 0.9554010000
O 2.8115630000 0.2199590000 0.0781090000
S 3.9431050000 -0.4600830000 -0.8369330000
O 3.5592670000 -0.1366010000 -2.1896550000
O 4.1010190000 -1.8408440000 -0.4284630000
C 5.4012610000 0.4383910000 -0.3966480000
C 5.4998580000 1.7760190000 -0.7764400000
C 6.3998860000 -0.1914860000 0.3326420000
C 6.6328200000 2.4853850000 -0.4146890000
H 4.7075780000 2.2495340000 -1.3449600000
C 7.5317700000 0.5384460000 0.6809780000
H 6.2958960000 -1.2312010000 0.6182180000
C 7.6629850000 1.8784550000 0.3160930000
H 6.7254260000 3.5271730000 -0.7021200000
H 8.3221420000 0.0581960000 1.2467230000
C 8.8872940000 2.6678360000 0.6865510000
H 9.4389740000 2.9570830000 -0.2118960000
H 8.6091140000 3.5863040000 1.2086090000
H 9.5514710000 2.0879540000 1.3275880000
F 0.5114800000 -1.2734840000 1.9278470000
H 3.8738180000 0.5784460000 1.8335670000
H 3.0001400000 -0.9763170000 1.7662600000
N -2.1538050000 -3.0346100000 -0.5685400000
C -3.1733760000 -3.2661350000 0.5397700000
C -2.9057010000 -2.7997130000 1.9965530000

H -3.3334920000 -4.3457480000 0.5575820000
H -4.0874000000 -2.8045800000 0.1719870000
O -2.3029500000 -1.5101430000 2.0327380000
C -2.1003150000 -3.8009040000 2.8315410000
H -2.1148390000 -3.4719770000 3.8732210000
H -1.0580020000 -3.8907290000 2.5251150000
H -2.5581850000 -4.7909950000 2.7793660000
C -4.2895190000 -2.6702880000 2.6361000000
H -4.8734020000 -1.8909900000 2.1442960000
H -4.1739830000 -2.4082050000 3.6890820000
H -4.8366450000 -3.6137960000 2.5670120000
H -1.3640740000 -1.6097820000 2.2297840000
C -2.0282570000 -1.5910260000 -1.0393110000
C -3.2535490000 -0.7134550000 -1.4291430000
C -0.8000310000 -3.4998730000 -0.0695860000
C 0.4875180000 -3.3583450000 -0.9137310000
C 0.4386930000 -3.8854350000 -2.3411610000
C 1.5452230000 -4.1318120000 -0.1180020000
O 0.8817520000 -1.9923250000 -1.0081070000
C -4.3004790000 -1.3774220000 -2.3144460000
O -2.6714800000 0.2948120000 -2.2490540000
C -3.9068250000 -0.0411520000 -0.2231340000
H -1.3997360000 -1.6495200000 -1.9246070000
H -1.4726280000 -1.0624670000 -0.2668490000
H -3.8375480000 -1.8637920000 -3.1755130000
H -4.9608760000 -0.5891650000 -2.6825980000
H -4.9107780000 -2.1018320000 -1.7724740000
H -4.4671890000 -0.7407900000 0.3963860000
H -4.5961140000 0.7252390000 -0.5887740000
H -3.1476960000 0.4343100000 0.4004710000
H -2.0694680000 0.8372760000 -1.7021220000
H -0.9423850000 -4.5459520000 0.2071050000
H -0.6097730000 -2.9172670000 0.8248290000
H 1.2131870000 -1.7141490000 -0.1451190000
H 0.0143000000 -4.8903940000 -2.3783130000
H 1.4600010000 -3.9225860000 -2.7258660000
H -0.1360830000 -3.2261830000 -2.9927330000
H 1.3388980000 -5.2040990000 -0.1253430000
H 1.5740220000 -3.7881050000 0.9215370000
H 2.5219930000 -3.9468440000 -0.5669140000
C -2.6092580000 -3.9184070000 -1.6984850000
H -3.6878940000 -3.8456910000 -1.7920710000
H -2.3368140000 -4.9440570000 -1.4563050000
H -2.1369990000 -3.6044320000 -2.6226330000
H 1.8460730000 0.6551460000 3.2330780000

Electronic Energy (Hartree)	Zero Point Correction (kcal/mol)	Electronic and Zero Point Correction (Hartree)	Gibbs Free Energy (kcal/mol)	Electronic and Gibbs Free Energy (Hartree)	Number of Imaginary Frequencies
−2800.932809	497.8208566	−2800.1395	425.4815615	−2800.254761	0

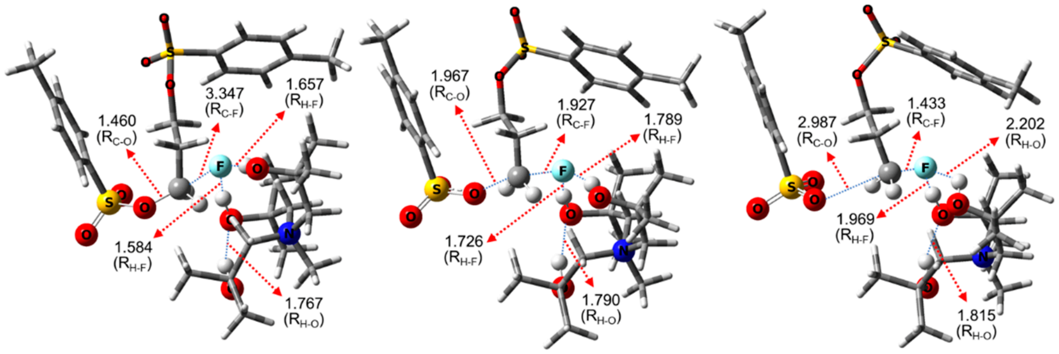

(pre-reaction complex) (transition state) (post-reaction complex)

SN2_2: pre-reaction complex
C -0.7224860000 -1.5525160000 1.1539420000
H -0.0920910000 -1.7982080000 2.0042740000
H -0.0912020000 -1.3901200000 0.2784220000
C -1.6004820000 -0.3392130000 1.4481580000
H -2.5820850000 -0.6637430000 1.8026920000
H -1.1282260000 0.2472490000 2.2366320000
C -1.7388190000 0.5332110000 0.2178950000
H -2.1163680000 -0.0132380000 -0.6464310000
H -0.7728490000 0.9707280000 -0.0446370000
O -1.4912450000 -2.7783940000 0.9596800000
S -2.2470880000 -3.0698700000 -0.4169870000
O -2.4375790000 -4.5031890000 -0.4195340000
O -1.5331210000 -2.4383650000 -1.5089510000
C -3.8094100000 -2.2640140000 -0.2075810000
C -4.5761020000 -2.5677590000 0.9169160000
C -4.2519150000 -1.3713340000 -1.1731710000
C -5.7981510000 -1.9370100000 1.0768380000
H -4.2165850000 -3.2714930000 1.6592010000
C -5.4841840000 -0.7499530000 -0.9940300000
H -3.6432370000 -1.1558910000 -2.0434460000
C -6.2676130000 -1.0182690000 0.1278960000
H -6.4019120000 -2.1562350000 1.9508920000
H -5.8367400000 -0.0450560000 -1.7386120000
C -7.5933710000 -0.3399000000 0.3309430000
H -7.8346830000 0.3134550000 -0.5075450000
H -8.3901220000 -1.0800100000 0.4384730000
H -7.5786510000 0.2593060000 1.2449850000
O -2.6844340000 1.5845620000 0.5484010000
S -2.8347690000 2.7697840000 -0.5180630000
O -3.8828820000 3.6054430000 0.0210620000
O -2.9746030000 2.1946230000 -1.8401260000
C -1.2804710000 3.6054040000 -0.4098170000
C -1.0467260000 4.4361340000 0.6847780000
C -0.3089360000 3.3764460000 -1.3752420000
C 0.1899300000 5.0493430000 0.8006740000
H -1.8195530000 4.6002650000 1.4266200000
C 0.9255070000 4.0025540000 -1.2395210000
H -0.5099120000 2.7181010000 -2.2116500000
C 1.1927480000 4.8370780000 -0.1539260000

H 0.3891400000 5.6954750000 1.6489240000
H 1.6950300000 3.8291090000 -1.9836020000
C 2.5345640000 5.4950330000 0.0029220000
H 3.2648840000 5.0689560000 -0.6860410000
H 2.4590090000 6.5674490000 -0.1985940000
H 2.9031160000 5.3789330000 1.0248180000
F 1.1139030000 0.2457280000 -0.9904930000
N 3.9990180000 -0.8133330000 0.7167750000
C 3.8169850000 0.0044490000 1.9940060000
C 2.5901450000 0.9618470000 2.1528870000
H 4.7303540000 0.5918220000 2.1043400000
H 3.7934520000 -0.7451510000 2.7848190000
O 1.3857370000 0.4493830000 1.6160230000
C 2.8567790000 2.3843450000 1.6422390000
H 2.0266350000 3.0195030000 1.9605010000
H 2.9224270000 2.4542780000 0.5556730000
H 3.7815710000 2.7806030000 2.0699270000
C 2.3675170000 1.0398160000 3.6647510000
H 2.0677990000 0.0615340000 4.0472180000
H 1.5747310000 1.7594920000 3.8766630000
H 3.2770200000 1.3570050000 4.1810410000
H 1.3515510000 0.4919680000 0.6305060000
C 2.8458950000 -1.8235240000 0.6434450000
C 3.0545310000 -3.2607680000 0.1060460000
C 4.0129320000 0.1930930000 -0.4185540000
C 4.0698800000 -0.2020560000 -1.9063640000
C 5.3465910000 -0.8963260000 -2.3667720000
C 3.9421160000 1.1382760000 -2.6453940000
O 2.9524120000 -1.0139000000 -2.2565160000
C 3.7558830000 -4.2042490000 1.0804120000
O 3.7796260000 -3.3047850000 -1.1093620000
C 1.6262000000 -3.7764080000 -0.1300700000
H 2.0845340000 -1.3342700000 0.0469920000
H 2.4621880000 -1.9146390000 1.6584210000
H 4.8188850000 -3.9912270000 1.1772340000
H 3.6560370000 -5.2262490000 0.7074800000
H 3.2923350000 -4.1519810000 2.0681340000
H 1.0409000000 -3.7782040000 0.7935440000
H 1.6795970000 -4.7982060000 -0.5112930000
H 1.1094360000 -3.1542890000 -0.8671020000
H 3.3845490000 -2.6332860000 -1.6977450000
H 4.8459110000 0.8609610000 -0.1930720000
H 3.0811550000 0.7331730000 -0.3192500000
H 2.1462460000 -0.5618660000 -1.8792610000
H 6.2208460000 -0.3203970000 -2.0544780000
H 5.3414170000 -0.9475320000 -3.4582310000
H 5.4338520000 -1.9107850000 -1.9838350000
H 4.7758070000 1.8059400000 -2.4143270000
H 3.0044370000 1.6294790000 -2.3731840000
H 3.9339880000 0.9448450000 -3.7196710000
C 5.3351380000 -1.4731180000 0.8514030000
H 5.3719690000 -1.9805060000 1.8133740000
H 6.0981390000 -0.6967690000 0.8140210000
H 5.4673810000 -2.1838080000 0.0443360000

Electronic Energy (Hartree)	Zero Point Correction (kcal/mol)	Electronic and Zero Point Correction (Hartree)	Gibbs Free Energy (kcal/mol)	Electronic and Gibbs Free Energy (Hartree)	Number of Imaginary frequencies
−2800.930218	498.3479646	−2800.136	428.5111773	−2800.247342	0

SN2_2: transition state
C -0.3888700000 -1.0448800000 0.3391320000
H 0.2794110000 -1.2524400000 1.1595690000
H -0.2604860000 -1.5830390000 -0.5860540000
C -1.4382830000 0.0178440000 0.4764940000
H -2.2234050000 -0.3246820000 1.1515080000
H -0.9768800000 0.9038130000 0.9175910000
C -2.0161450000 0.3521360000 -0.8836280000
H -2.4889670000 -0.5127760000 -1.3483930000
H -1.2348380000 0.7225390000 -1.5491150000
O -1.4100700000 -2.5892740000 1.0045750000
S -2.3814620000 -3.2844500000 0.0754550000
O -2.5736880000 -4.6746040000 0.4849750000
O -2.0156200000 -3.0778260000 -1.3306260000
C -3.9299170000 -2.4314740000 0.3316020000
C -4.3544870000 -2.1764140000 1.6347690000
C -4.7078140000 -2.0609390000 -0.7559440000
C -5.5655350000 -1.5332270000 1.8385270000
H -3.7346470000 -2.4639180000 2.4767920000
C -5.9223940000 -1.4151400000 -0.5353570000
H -4.3607190000 -2.2623470000 -1.7626750000
C -6.3667420000 -1.1429200000 0.7577150000
H -5.8985920000 -1.3257610000 2.8502730000
H -6.5300250000 -1.1172470000 -1.3829990000
C -7.6737370000 -0.4390490000 1.0020670000
H -8.1915600000 -0.2329320000 0.0649280000
H -8.3268380000 -1.0468630000 1.6332480000
H -7.5085210000 0.5089100000 1.5206920000
O -3.0584000000 1.3489700000 -0.7172170000
S -2.8399860000 2.7826850000 -1.3976430000
O -4.0492180000 3.5067350000 -1.0833790000
O -2.4475440000 2.5906970000 -2.7780300000
C -1.4794960000 3.4725440000 -0.5042110000
C -1.7208960000 4.0294770000 0.7500670000
C -0.2053140000 3.4423030000 -1.0579570000
C -0.6598540000 4.5877720000 1.4447370000
H -2.7221070000 4.0395380000 1.1646100000
C 0.8454070000 4.0077340000 -0.3433680000
H -0.0392780000 3.0007590000 -2.0336770000
C 0.6319820000 4.5989960000 0.9035830000
H -0.8348900000 5.0368190000 2.4163200000
H 1.8430200000 4.0025060000 -0.7696980000
C 1.7515970000 5.2831920000 1.6368710000
H 2.7248570000 4.9715320000 1.2563570000
H 1.6684880000 6.3664480000 1.5068840000
H 1.7044650000 5.0767860000 2.7076420000
F 0.9032500000 0.1990620000 -0.3641470000
N 4.3403570000 -0.6384390000 0.3621170000
C 4.4715200000 -0.0133680000 1.7504580000

C 3.2683040000 0.7430890000 2.3966850000
H 5.3185280000 0.6723370000 1.6899490000
H 4.7529530000 -0.8517470000 2.3868350000
O 2.0083310000 0.1529280000 2.1189680000
C 3.2432060000 2.2396050000 2.0712150000
H 2.4563390000 2.7029940000 2.6705400000
H 3.0300880000 2.4535580000 1.0229320000
H 4.1967850000 2.7065290000 2.3302940000
C 3.4756620000 0.5826460000 3.9024100000
H 3.3785040000 -0.4688880000 4.1810120000
H 2.7200850000 1.1614370000 4.4363320000
H 4.4656570000 0.9363180000 4.2003760000
H 1.6891760000 0.3944370000 1.2309970000
C 3.3191110000 -1.7813050000 0.4516330000
C 3.5274580000 -3.1160610000 -0.3070430000
C 3.9140840000 0.4781060000 -0.5708390000
C 3.6227350000 0.2632000000 -2.0688170000
C 4.8083730000 -0.1618530000 -2.9261840000
C 3.1206680000 1.6309070000 -2.5500550000
O 2.5742270000 -0.6886080000 -2.2576950000
C 4.5406570000 -4.0495210000 0.3494000000
O 3.9330950000 -2.9471750000 -1.6543600000
C 2.1446210000 -3.7818930000 -0.2702240000
H 2.3772750000 -1.3437850000 0.1401800000
H 3.2324550000 -2.0272650000 1.5081360000
H 5.5638360000 -3.6901980000 0.2549040000
H 4.4840230000 -5.0236070000 -0.1415930000
H 4.3082850000 -4.1830900000 1.4081350000
H 1.7798270000 -3.8965370000 0.7541010000
H 2.2120800000 -4.7693340000 -0.7306800000
H 1.4214130000 -3.1842390000 -0.8323330000
H 3.3237660000 -2.3016910000 -2.0541620000
H 4.6843140000 1.2448960000 -0.4689600000
H 2.9863910000 0.8538470000 -0.1567660000
H 1.8242900000 -0.4060730000 -1.6931140000
H 5.6597150000 0.4972450000 -2.7426530000
H 4.5261980000 -0.0761360000 -3.9778930000
H 5.1095420000 -1.1909610000 -2.7422600000
H 3.8863610000 2.4017230000 -2.4341950000
H 2.2306790000 1.9256410000 -1.9871670000
H 2.8554300000 1.5541470000 -3.6060040000
C 5.7198720000 -1.1158020000 0.0202460000
H 6.0988830000 -1.6904880000 0.8626200000
H 6.3459720000 -0.2392240000 -0.1386550000
H 5.6805240000 -1.7327080000 -0.8688000000

Electronic Energy (Hartree)	Zero Point Correction (kcal/mol)	Electronic and Zero Point Correction (Hartree)	Gibbs Free Energy (kcal/mol)	Electronic and Gibbs Free Energy (Hartree)	Number of Imaginary Frequencies
−2800.889065	497.7888536	−2800.0958	428.2099728	−2800.206669	1

S_N2_2: post-reaction complex
C 0.0179380000 -0.5567740000 0.2767700000
H 0.5193040000 -0.7151840000 1.2303440000

H -0.0416120000 -1.4865860000 -0.2864340000
C -1.3249190000 0.1066150000 0.4370900000
H -1.9081900000 -0.4831460000 1.1463670000
H -1.1831170000 1.1083660000 0.8534560000
C -2.0450390000 0.1763490000 -0.8959930000
H -2.3282850000 -0.8178570000 -1.2401540000
H -1.4271290000 0.6470080000 -1.6641800000
O -1.2854840000 -2.9487360000 1.5010350000
S -2.0943490000 -3.4951190000 0.3935280000
O -2.3873720000 -4.9316360000 0.5359160000
O -1.5649240000 -3.1422590000 -0.9416190000
C -3.6775460000 -2.6520190000 0.4980010000
C -4.1025580000 -2.1069540000 1.7031890000
C -4.4924030000 -2.5850060000 -0.6295480000
C -5.3442120000 -1.4808590000 1.7751600000
H -3.4561930000 -2.1587950000 2.5719240000
C -5.7301410000 -1.9610120000 -0.5456190000
H -4.1486020000 -3.0042590000 -1.5687060000
C -6.1729590000 -1.3965870000 0.6553810000
H -5.6725690000 -1.0487620000 2.7148280000
H -6.3626010000 -1.9031410000 -1.4257470000
C -7.4946770000 -0.6794670000 0.7207120000
H -8.2510160000 -1.1940810000 0.1249040000
H -7.8510560000 -0.6019820000 1.7488400000
H -7.3955050000 0.3348580000 0.3220940000
O -3.2875610000 0.9178040000 -0.7423330000
S -3.3785190000 2.3772450000 -1.3919380000
O -4.6993750000 2.8404990000 -1.0353670000
O -2.9909730000 2.2960430000 -2.7851230000
C -2.1540040000 3.2959010000 -0.5090180000
C -2.4625810000 3.7679860000 0.7653740000
C -0.8942190000 3.4674520000 -1.0675960000
C -1.4829000000 4.4370170000 1.4801070000
H -3.4525520000 3.6218050000 1.1815040000
C 0.0765640000 4.1395680000 -0.3312490000
H -0.6784500000 3.0955900000 -2.0621840000
C -0.2034150000 4.6345400000 0.9434950000
H -1.7104970000 4.8211730000 2.4686450000
H 1.0606170000 4.2924170000 -0.7607570000
C 0.8294010000 5.3932750000 1.7292280000
H 1.7760430000 5.4469650000 1.1911830000
H 0.4817860000 6.4116320000 1.9218500000
H 1.0017200000 4.9193140000 2.6987800000
F 0.8560090000 0.3278140000 -0.4781940000
N 4.5732430000 -0.3250260000 0.2030200000
C 4.8312480000 0.5295630000 1.4430850000
C 3.6984730000 1.4147060000 2.0431030000
H 5.6760060000 1.1726060000 1.1904860000
H 5.1630380000 -0.1922940000 2.1888760000
O 2.4122160000 0.8155320000 1.8986740000
C 3.6911950000 2.8376440000 1.4847520000
H 2.9406500000 3.4177530000 2.0261510000
H 3.4533510000 2.8789560000 0.4202950000
H 4.6646990000 3.3087990000 1.6356090000

C 3.9841820000 1.4730430000 3.5426950000
H 3.8612410000 0.4807810000 3.9823080000
H 3.2851890000 2.1620720000 4.0199340000
H 5.0041780000 1.8168550000 3.7319290000
H 1.9227430000 1.2693400000 1.2021840000
C 3.5768170000 -1.4334150000 0.5719640000
C 3.7800050000 -2.8904270000 0.0869400000
C 4.0298530000 0.5979130000 -0.8675760000
C 3.6924500000 0.1217900000 -2.2948020000
C 4.8642740000 -0.3974660000 -3.1184100000
C 3.1068910000 1.3623060000 -2.9785110000
O 2.6983930000 -0.9084320000 -2.2899200000
C 4.8453260000 -3.6519060000 0.8698810000
O 4.1242840000 -2.9907810000 -1.2857410000
C 2.4183960000 -3.5573270000 0.3204350000
H 2.6145300000 -1.0746330000 0.2234740000
H 3.5354370000 -1.4607230000 1.6586740000
H 5.8520000000 -3.2862460000 0.6751290000
H 4.8091980000 -4.7025850000 0.5738010000
H 4.6474120000 -3.5883550000 1.9420190000
H 2.0983160000 -3.4644080000 1.3615190000
H 2.4927900000 -4.6172110000 0.0698230000
H 1.6564700000 -3.1014430000 -0.3178510000
H 3.4726540000 -2.4704760000 -1.7835000000
H 4.7554390000 1.4095910000 -0.9452250000
H 3.1066560000 0.9912300000 -0.4535170000
H 1.8831150000 -0.5505940000 -1.9101640000
H 5.7104530000 0.2889170000 -3.0504950000
H 4.5530430000 -0.4621240000 -4.1630430000
H 5.1827240000 -1.3888740000 -2.8021590000
H 3.8536630000 2.1549250000 -3.0590740000
H 2.2471550000 1.7456980000 -2.4207820000
H 2.7777070000 1.0863140000 -3.9815820000
C 5.9312630000 -0.8433090000 -0.1768260000
H 6.4144300000 -1.2158280000 0.7233400000
H 6.5020530000 -0.0085390000 -0.5799280000
H 5.8308300000 -1.6371480000 -0.9050790000

Electronic Energy (Hartree)	Zero Point Correction (kcal/mol)	Electronic and Zero Point Correction (Hartree)	Gibbs Free Energy (kcal/mol)	Electronic and Gibbs Free Energy (Hartree)	Number of Imaginary Frequencies
−2800.928004	497.8967853	−2800.1346	426.1335438	−2800.248917	0

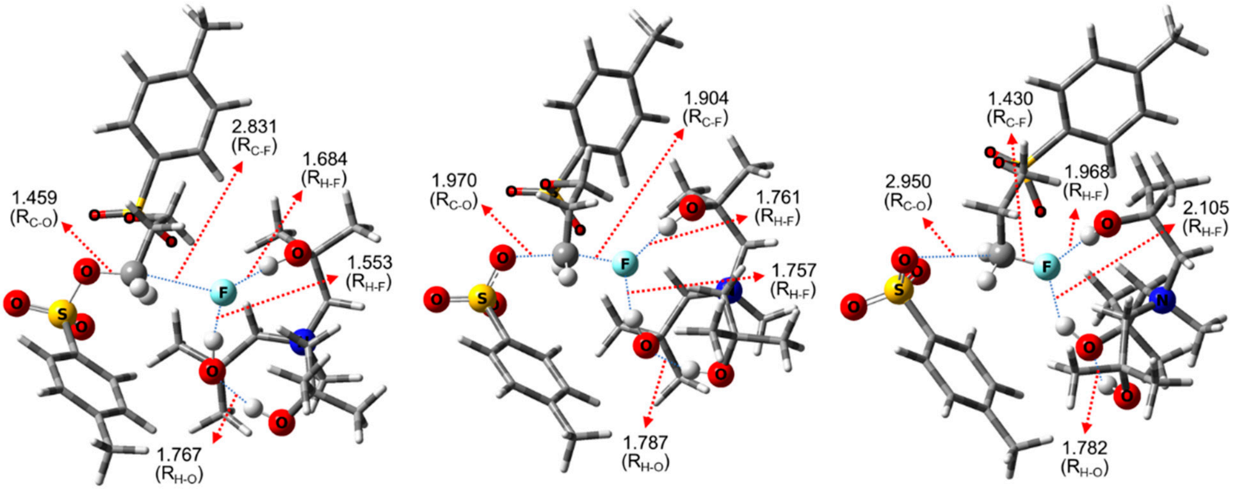

(pre-reaction complex) (transition state) (post-reaction complex)

SN2_3: pre-reaction complex
C -0.4223260000 -2.0338970000 -1.2148510000
H -1.2760950000 -2.0111080000 -1.8935540000
H -0.6254700000 -1.3940680000 -0.3564600000
C 0.8544280000 -1.6077940000 -1.9140320000
H 1.3119030000 -2.4674610000 -2.4096910000
H 0.5930920000 -0.8735190000 -2.6765560000
C 1.8366430000 -0.9333730000 -0.9868040000
H 1.3542080000 -0.0867740000 -0.4958500000
H 2.7301390000 -0.6034490000 -1.5239090000
O -0.2639420000 -3.4149050000 -0.7706140000
S -1.1424510000 -3.8873340000 0.4772490000
O -0.8552590000 -3.0344160000 1.6121010000
O -0.8992680000 -5.3094750000 0.5698110000
C -2.8054130000 -3.5880070000 -0.0450740000
C -3.3872890000 -2.3528090000 0.2283120000
C -3.4723350000 -4.5668000000 -0.7731080000
C -4.6717010000 -2.1070270000 -0.2356340000
H -2.8492500000 -1.5894200000 0.7802190000
C -4.7600470000 -4.3020740000 -1.2211780000
H -2.9979530000 -5.5196550000 -0.9760930000
C -5.3754500000 -3.0746150000 -0.9603050000
H -5.1360190000 -1.1474160000 -0.0337240000
H -5.2949120000 -5.0588440000 -1.7841920000
C -6.7779460000 -2.8015770000 -1.4273880000
H -7.0613760000 -3.4740400000 -2.2376420000
H -7.4837280000 -2.9474970000 -0.6043760000
H -6.8807520000 -1.7703820000 -1.7698890000
O 2.2345740000 -1.9042280000 0.0205230000
S 3.1578290000 -1.3838550000 1.2161760000
O 3.3562900000 -2.5477060000 2.0495670000
O 2.5792760000 -0.1823830000 1.7838880000
C 4.6654930000 -0.9539600000 0.3975020000
C 5.4007760000 -1.9599560000 -0.2278010000
C 5.0730370000 0.3720110000 0.3746020000
C 6.5693450000 -1.6149260000 -0.8855920000

H 5.0657220000 -2.9905840000 -0.2002380000
C 6.2529310000 0.6962300000 -0.2883090000
H 4.4821820000 1.1335880000 0.8691210000
C 7.0114950000 -0.2855060000 -0.9254030000
H 7.1524320000 -2.3853960000 -1.3784710000
H 6.5848650000 1.7281130000 -0.3115230000
C 8.2875970000 0.0600720000 -1.6405660000
H 8.4565630000 1.1369290000 -1.6483240000
H 9.1394080000 -0.4208330000 -1.1524680000
H 8.2583180000 -0.2977140000 -2.6724380000
F -0.7167180000 0.7816570000 -1.2252960000
N -1.0570010000 3.7170660000 0.6395080000
C 0.2510040000 4.4047850000 0.2483010000
C 1.2612560000 3.6968210000 -0.7127580000
H 0.7612110000 4.6275010000 1.1870760000
H -0.0706530000 5.3450270000 -0.1990870000
O 0.6474610000 2.9911180000 -1.7712680000
C 2.2904160000 2.8205870000 0.0154830000
H 3.0277110000 2.4883520000 -0.7201480000
H 1.8707170000 1.9317890000 0.4863230000
H 2.8099240000 3.3982500000 0.7852510000
C 2.0183580000 4.8504170000 -1.3751410000
H 1.3363310000 5.4325960000 -1.9985280000
H 2.8126510000 4.4458350000 -2.0050330000
H 2.4648470000 5.5109780000 -0.6277130000
H 0.2450590000 2.1393420000 -1.4871590000
C -1.9132030000 3.5863100000 -0.6274160000
C -3.4540350000 3.7375320000 -0.5890100000
C -0.6521690000 2.3921010000 1.2573910000
C -1.6565340000 1.3260790000 1.7313520000
C -2.5826350000 1.7257480000 2.8754260000
C -0.7650690000 0.1739910000 2.2131530000
O -2.4479610000 0.8655000000 0.6359550000
C -3.9420430000 5.1803510000 -0.4855360000
O -4.0662000000 3.0156690000 0.4638170000
C -3.9127180000 3.1603950000 -1.9363510000
H -1.6661600000 2.6126660000 -1.0367600000
H -1.5378460000 4.3385800000 -1.3187620000
H -3.7743930000 5.6093700000 0.5007270000
H -5.0177100000 5.1986120000 -0.6757490000
H -3.4498410000 5.8066510000 -1.2329480000
H -3.4562540000 3.6899990000 -2.7767690000
H -4.9978120000 3.2534770000 -2.0113090000
H -3.6454170000 2.1021530000 -2.0068720000
H -3.6813250000 2.1181180000 0.4545790000
H 0.0023690000 2.6577090000 2.0894930000
H -0.0743960000 1.8960130000 0.4881990000
H -1.8238950000 0.7260090000 -0.1372290000
H -2.0019040000 2.1557700000 3.6947450000
H -3.0825000000 0.8272040000 3.2460040000
H -3.3481970000 2.4356130000 2.5713260000
H -0.1952820000 0.4599740000 3.1007590000
H -0.0567160000 -0.1305330000 1.4385900000
H -1.3945650000 -0.6828390000 2.4580530000

C -1.6858950000 4.6061010000 1.6651570000
H -1.7244590000 5.6178580000 1.2658050000
H -1.0616730000 4.5859070000 2.5570280000
H -2.6852400000 4.2495460000 1.8856960000

Electronic Energy (Hartree)	Zero Point Correction (kcal/mol)	Electronic and Zero Point Correction (Hartree)	Gibbs Free Energy (kcal/mol)	Electronic and Gibbs Free Energy (Hartree)	Number of Imaginary Frequencies
−2800.927972	498.2726635	−2800.1339	427.9539489	−2800.245985	0

SN2_3: transition state
C -0.6896050000 -1.5748040000 -1.0930700000
H -1.7028760000 -1.4103910000 -1.4151310000
H -0.4681580000 -1.6684280000 -0.0397010000
C 0.4145560000 -1.6171220000 -2.1063670000
H 0.4226930000 -2.5918750000 -2.5942240000
H 0.2202950000 -0.8600500000 -2.8677470000
C 1.7637520000 -1.3389270000 -1.4850800000
H 1.7243420000 -0.4334990000 -0.8792550000
H 2.5457200000 -1.2480740000 -2.2397630000
O -1.0962570000 -3.5024000000 -1.1209110000
S -1.9184970000 -3.9863560000 0.0577170000
O -1.2838230000 -3.6250220000 1.3277600000
O -2.2731380000 -5.3935570000 -0.1138710000
C -3.4305470000 -3.0390460000 -0.0396370000
C -3.5056480000 -1.8033130000 0.5965190000
C -4.4781410000 -3.4962350000 -0.8308510000
C -4.6436490000 -1.0225030000 0.4361700000
H -2.6817920000 -1.4535080000 1.2088810000
C -5.6146870000 -2.7085550000 -0.9754150000
H -4.4070680000 -4.4607240000 -1.3201520000
C -5.7130610000 -1.4631370000 -0.3492050000
H -4.6931370000 -0.0540700000 0.9225190000
H -6.4364390000 -3.0632040000 -1.5885790000
C -6.9528120000 -0.6227250000 -0.4929250000
H -7.4926290000 -0.8705440000 -1.4079720000
H -7.6281600000 -0.7938900000 0.3506470000
H -6.7054640000 0.4403870000 -0.5064710000
O 2.1018190000 -2.4739100000 -0.6401800000
S 2.7614890000 -2.1979050000 0.7873150000
O 2.9678100000 -3.5148810000 1.3450460000
O 1.9602070000 -1.2362270000 1.5193740000
C 4.3044320000 -1.4380750000 0.3742410000
C 5.0474220000 -1.9356680000 -0.6950460000
C 4.7671030000 -0.3941110000 1.1636950000
C 6.2720520000 -1.3535370000 -0.9789370000
H 4.6692930000 -2.7525600000 -1.2997320000
C 6.0037790000 0.1689230000 0.8672890000
H 4.1679310000 -0.0186640000 1.9847330000
C 6.7679680000 -0.2977710000 -0.2027910000
H 6.8575990000 -1.7218560000 -1.8143960000
H 6.3754930000 0.9871170000 1.4739760000
C 8.1024270000 0.3116570000 -0.5299560000
H 8.3444940000 1.1234700000 0.1559380000

H 8.8911210000 -0.4424760000 -0.4689350000
H 8.1044270000 0.7040540000 -1.5498660000
F -0.5873170000 0.3097040000 -0.8398610000
N -0.2649130000 3.6170980000 0.4596970000
C 1.1289230000 3.9442930000 -0.0789870000
C 1.9284270000 2.9018010000 -0.9211130000
H 1.7265070000 4.2128820000 0.7937780000
H 0.9702460000 4.8411020000 -0.6774310000
O 1.1293980000 2.1727010000 -1.8372540000
C 2.7940040000 1.9631240000 -0.0720650000
H 3.4316420000 1.3841090000 -0.7456780000
H 2.2223520000 1.2564940000 0.5320230000
H 3.4381200000 2.5398260000 0.5971870000
C 2.8632690000 3.7504740000 -1.7849950000
H 2.2792750000 4.3565330000 -2.4808770000
H 3.5219220000 3.0934010000 -2.3555970000
H 3.4758280000 4.4122130000 -1.1679100000
H 0.6062480000 1.4787350000 -1.3959330000
C -1.2084440000 3.4563460000 -0.7398050000
C -2.6640490000 3.9884270000 -0.7032800000
C -0.1184150000 2.3533810000 1.2853450000
C -1.3049100000 1.6549960000 1.9787030000
C -2.0004810000 2.4462470000 3.0796320000
C -0.6931440000 0.3865330000 2.5841500000
O -2.2949840000 1.2595030000 1.0239100000
C -2.7708010000 5.4960850000 -0.9131060000
O -3.3521440000 3.6888910000 0.4986060000
C -3.3529310000 3.2749030000 -1.8746390000
H -1.2253180000 2.3917320000 -0.9493810000
H -0.7151430000 3.9428730000 -1.5788030000
H -2.3878680000 6.0650660000 -0.0675190000
H -3.8241080000 5.7561040000 -1.0398230000
H -2.2304960000 5.7951580000 -1.8138190000
H -2.8396210000 3.4682150000 -2.8201610000
H -4.3812150000 3.6326880000 -1.9539920000
H -3.3699210000 2.1944040000 -1.7034140000
H -3.2325920000 2.7366640000 0.6609880000
H 0.6362900000 2.5906720000 2.0368600000
H 0.2820630000 1.6160340000 0.5998350000
H -1.8295040000 0.7702520000 0.3144220000
H -1.2624100000 2.8310380000 3.7867290000
H -2.6736910000 1.7747680000 3.6175040000
H -2.5895210000 3.2757440000 2.6945660000
H 0.0618120000 0.6294640000 3.3359460000
H -0.2231380000 -0.2246930000 1.8098630000
H -1.4876560000 -0.1907430000 3.0616090000
C -0.6217470000 4.7811990000 1.3346870000
H -0.4270230000 5.6971060000 0.7807580000
H 0.0175900000 4.7423010000 2.2154020000
H -1.6673340000 4.7218480000 1.6096510000

Electronic Energy (Hartree)	Zero Point Correction (kcal/mol)	Electronic and Zero Point Correction (Hartree)	Gibbs Free Energy (kcal/mol)	Electronic and Gibbs Free Energy (Hartree)	Number of Imaginary Frequencies

−2800.889089	497.7179451	−2800.0959	428.2538984	−2800.206623	1

S_N2_3: post-reaction complex
C -0.6144560000 -1.4157680000 -0.9791210000
H -1.6387230000 -1.4066590000 -1.3421030000
H -0.4984000000 -2.1442340000 -0.1782350000
C 0.3723100000 -1.6071590000 -2.1016510000
H 0.1644640000 -2.5804710000 -2.5521600000
H 0.2209500000 -0.8420950000 -2.8691100000
C 1.8167900000 -1.5434870000 -1.6538010000
H 2.0286020000 -0.6276070000 -1.0986050000
H 2.5000100000 -1.6021270000 -2.5003940000
O -1.8908250000 -3.9908040000 -1.6447890000
S -2.5074280000 -4.2380260000 -0.3246230000
O -1.5222420000 -4.3041060000 0.7716870000
O -3.4603800000 -5.3608280000 -0.3335960000
C -3.4962670000 -2.7733550000 0.0048920000
C -3.2556420000 -1.9889080000 1.1274820000
C -4.4814990000 -2.4079800000 -0.9071430000
C -4.0107690000 -0.8384250000 1.3369370000
H -2.4767800000 -2.2797800000 1.8233760000
C -5.2252030000 -1.2526030000 -0.6925090000
H -4.6619090000 -3.0171430000 -1.7863000000
C -5.0022930000 -0.4515560000 0.4311630000
H -3.8186200000 -0.2230730000 2.2094500000
H -5.9892700000 -0.9658260000 -1.4077490000
C -5.8077680000 0.7973040000 0.6729090000
H -6.4453930000 1.0263230000 -0.1822920000
H -6.4470090000 0.6783160000 1.5522370000
H -5.1553090000 1.6554410000 0.8550450000
O 2.1121190000 -2.6961830000 -0.8144020000
S 2.5701530000 -2.4426330000 0.6957860000
O 2.8293080000 -3.7660800000 1.2147960000
O 1.6000000000 -1.5971160000 1.3630940000
C 4.0717260000 -1.5241710000 0.5043350000
C 4.9863260000 -1.9060610000 -0.4758950000
C 4.3254420000 -0.4696380000 1.3708090000
C 6.1710760000 -1.1968640000 -0.5890840000
H 4.7701070000 -2.7311990000 -1.1453850000
C 5.5254210000 0.2226100000 1.2456860000
H 3.5966030000 -0.1860650000 2.1209040000
C 6.4587630000 -0.1273950000 0.2692800000
H 6.8880810000 -1.4745600000 -1.3540240000
H 5.7347660000 1.0503140000 1.9141330000
C 7.7563220000 0.6183640000 0.1303650000
H 7.8010800000 1.4652590000 0.8151770000
H 8.5994760000 -0.0440610000 0.3432350000
H 7.8802430000 0.9863180000 -0.8908340000
F -0.3909530000 -0.1345440000 -0.3841690000
N -0.1452270000 3.5507850000 0.0166380000
C 1.1097620000 3.8220110000 -0.8196050000
C 1.9028190000 2.6628730000 -1.4876750000
H 1.7892630000 4.3661960000 -0.1616140000
H 0.7600180000 4.5022580000 -1.5956690000

O 1.0567990000 1.7092800000 -2.1221220000
C 2.9077260000 1.9646880000 -0.5640870000
H 3.5649470000 1.3391500000 -1.1745260000
H 2.4392250000 1.3167920000 0.1779980000
H 3.5217810000 2.6993360000 -0.0372630000
C 2.6761790000 3.3357080000 -2.6214050000
H 1.9818010000 3.7527620000 -3.3535620000
H 3.3070370000 2.5943280000 -3.1141860000
H 3.3101340000 4.1380090000 -2.2372530000
H 0.6822840000 1.1152020000 -1.4600450000
C -1.2432250000 3.0064940000 -0.9075190000
C -2.7182790000 3.4673060000 -0.7803970000
C 0.2499790000 2.5563570000 1.0885470000
C -0.7133700000 2.0747320000 2.1895670000
C -1.2290160000 3.1443650000 3.1439770000
C 0.0963060000 1.0384790000 2.9747390000
O -1.8624680000 1.4266530000 1.6313720000
C -2.9817650000 4.8402440000 -1.3904420000
O -3.1909080000 3.5100170000 0.5564040000
C -3.5046050000 2.4101800000 -1.5677110000
H -1.2127780000 1.9280040000 -0.7849560000
H -0.9273180000 3.2300250000 -1.9240450000
H -2.5108030000 5.6458230000 -0.8294320000
H -4.0592770000 5.0186640000 -1.3918040000
H -2.6245270000 4.8727240000 -2.4218080000
H -3.1476680000 2.3333120000 -2.5980230000
H -4.5608820000 2.6859070000 -1.5820310000
H -3.4039730000 1.4296100000 -1.0908450000
H -2.9666910000 2.6576290000 0.9671870000
H 1.1313370000 2.9853270000 1.5686420000
H 0.5520030000 1.6634290000 0.5507500000
H -1.5887420000 0.6169790000 1.1748720000
H -0.4049070000 3.7689660000 3.4941370000
H -1.6806330000 2.6508810000 4.0068970000
H -1.9864290000 3.7776060000 2.6858530000
H 0.9506280000 1.5053230000 3.4703870000
H 0.4619070000 0.2448440000 2.3162570000
H -0.5489880000 0.5957110000 3.7357750000
C -0.4862020000 4.8827010000 0.6207770000
H -0.4383270000 5.6333630000 -0.1649930000
H 0.2585850000 5.1011170000 1.3843540000
H -1.4811520000 4.8466610000 1.0449380000

Electronic Energy (Hartree)	Zero Point Correction (kcal/mol)	Electronic and Zero Point Correction (Hartree)	Gibbs Free Energy (kcal/mol)	Electronic and Gibbs Free Energy (Hartree)	Number of Imaginary Frequencies
−2800.929628	498.145279	−2800.1358	427.4205658	−2800.24849	0

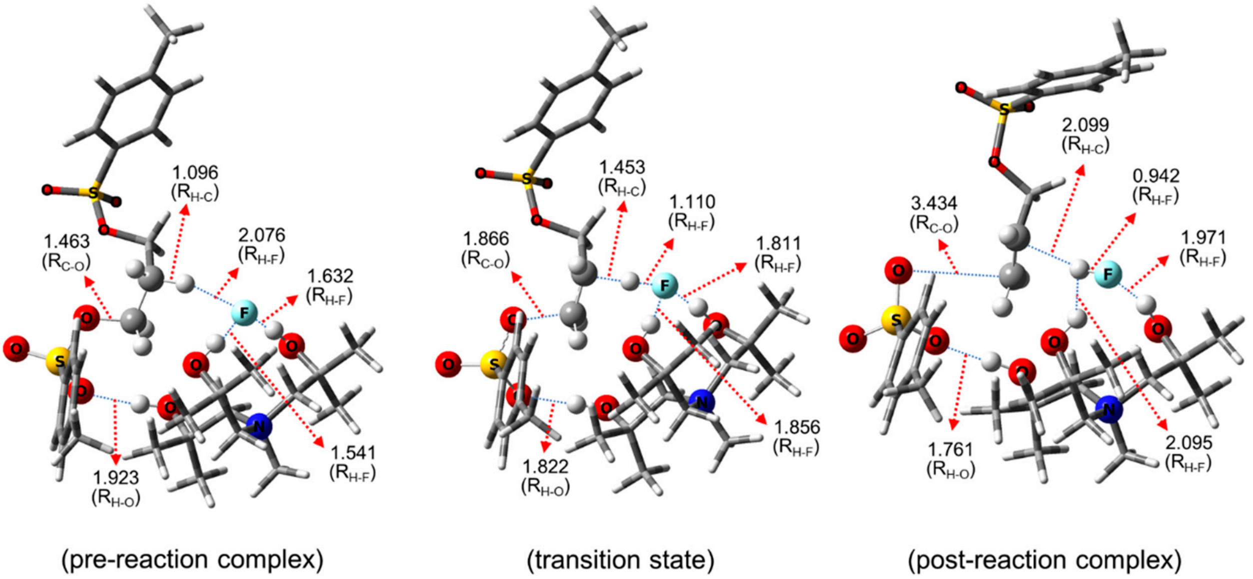

Figure S2. Pre- and post-reaction complexes and the transition states of E2 reactions using TBMA-^{18}F in CH_3CN.

E2_1: pre-reaction complex
C -0.4187650000 -0.6616330000 -0.5993030000
H 0.5511180000 -0.7491120000 -0.1012310000
H -0.2941660000 -0.1604630000 -1.5604980000
C -1.3923720000 0.0695880000 0.2948620000
H -1.6168130000 -0.5323240000 1.1796920000
H -0.8794810000 0.9795130000 0.6261940000
C -2.6711560000 0.5043630000 -0.3819630000
O -0.9478670000 -2.0018540000 -0.8543740000
S 0.0547830000 -3.0985000000 -1.4110130000
O 0.9041380000 -2.5035750000 -2.4347290000
O -0.7719730000 -4.2232640000 -1.7790370000
C 1.0615790000 -3.4805850000 -0.0053180000
C 2.3254680000 -4.0178420000 -0.2341260000
C 0.5736070000 -3.2820500000 1.2812830000
C 3.1189720000 -4.3406920000 0.8564190000
H 2.6885220000 -4.1669220000 -1.2444450000
C 1.3877810000 -3.6069390000 2.3590060000
H -0.4144550000 -2.8673160000 1.4429850000
C 2.6678620000 -4.1292650000 2.1639820000
H 4.1085230000 -4.7534590000 0.6923820000
H 1.0252420000 -3.4419500000 3.3675630000
C 3.5685570000 -4.4115170000 3.3327630000
H 2.9978720000 -4.5066170000 4.2571180000
H 4.1434060000 -5.3256750000 3.1739500000
H 4.2816320000 -3.5901240000 3.4584530000
O -3.4796670000 -0.6748170000 -0.6459350000
S -4.8725260000 -0.4584380000 -1.4013800000
O -5.3796090000 -1.7955880000 -1.6046850000
O -4.6628630000 0.4162270000 -2.5370410000
C -5.8616450000 0.3812200000 -0.2007990000
C -6.3181420000 -0.3344520000 0.9049840000
C -6.1383730000 1.7316620000 -0.3628820000

C -7.0732710000 0.3280160000 1.8581140000
H -6.0917170000 -1.3890390000 1.0119870000
C -6.9010350000 2.3770310000 0.6051910000
H -5.7699790000 2.2658920000 -1.2302010000
C -7.3755240000 1.6895310000 1.7222960000
H -7.4380150000 -0.2149550000 2.7233870000
H -7.1277520000 3.4308580000 0.4893450000
C -8.2068370000 2.3825400000 2.7653420000
H -7.7800470000 2.2321950000 3.7596740000
H -8.2723240000 3.4530790000 2.5704570000
H -9.2196080000 1.9709800000 2.7788850000
F 0.5672990000 2.4341850000 0.9445810000
H -3.2398000000 1.1721450000 0.2707240000
H -2.4658070000 1.0056480000 -1.3299990000
N 3.9713600000 2.0253700000 -0.0368580000
C 4.2500530000 1.0756200000 1.1206930000
C 3.1218520000 0.6073730000 2.0973400000
H 5.0236610000 1.5741410000 1.7084580000
H 4.7036040000 0.2042470000 0.6604690000
O 1.8921700000 0.3336890000 1.4629230000
C 2.9167770000 1.5551460000 3.2877200000
H 2.3012750000 1.0433550000 4.0312410000
H 2.4009280000 2.4767130000 3.0181640000
H 3.8725400000 1.8132750000 3.7502690000
C 3.6369630000 -0.7291590000 2.6404460000
H 3.7176030000 -1.4579820000 1.8298170000
H 2.9321850000 -1.1107130000 3.3824500000
H 4.6167190000 -0.6122730000 3.1117880000
H 1.3685970000 1.1743200000 1.3259940000
C 3.0133020000 1.4758240000 -1.0794040000
C 3.3255530000 0.2188210000 -1.9324700000
C 3.3852160000 3.2908570000 0.5656670000
C 2.7831420000 4.4123120000 -0.3092320000
C 3.6638420000 4.9439280000 -1.4342000000
C 2.4981540000 5.5454080000 0.6888710000
O 1.5600020000 3.9979020000 -0.8930000000
C 4.4184030000 0.4203420000 -2.9809890000
O 2.0867580000 0.1203220000 -2.6544980000
C 3.5546040000 -1.0725420000 -1.1551220000
H 2.8586390000 2.2910010000 -1.7816600000
H 2.0768150000 1.3027610000 -0.5612220000
H 4.2605460000 1.3567860000 -3.5222210000
H 4.3528850000 -0.4027530000 -3.6965950000
H 5.4229400000 0.4178360000 -2.5587890000
H 4.5724540000 -1.1413860000 -0.7679950000
H 3.4154330000 -1.9137960000 -1.8394300000
H 2.8454810000 -1.1651320000 -0.3287080000
H 1.7553840000 -0.7855370000 -2.5881860000
H 4.1751890000 3.6933740000 1.2011590000
H 2.5586060000 2.9545520000 1.1798350000
H 1.0838830000 3.4474700000 -0.2257010000
H 4.6589850000 5.2084580000 -1.0695640000
H 3.1950270000 5.8414980000 -1.8445710000
H 3.7594610000 4.2226710000 -2.2475780000

H 3.4181170000 5.9647060000 1.1037880000
H 1.8735290000 5.1810180000 1.5097050000
H 1.9531870000 6.3343040000 0.1671510000
C 5.3077340000 2.3213700000 -0.6457770000
H 5.8481820000 1.3872630000 -0.7742460000
H 5.8582390000 2.9688300000 0.0342400000
H 5.1696980000 2.8064160000 -1.6067760000

Electronic Energy (Hartree)	Zero Point Correction (kcal/mol)	Electronic and Zero Point Correction (Hartree)	Gibbs Free Energy (kcal/mol)	Electronic and Gibbs Free Energy (Hartree)	Number of Imaginary Frequencies
−2800.922818	497.7995213	−2800.1295	426.4397685	−2800.243243	0

E2_1: transition state
C -0.2502170000 -0.6578080000 -0.8511720000
H 0.6987630000 -0.8205190000 -0.3471640000
H -0.1698140000 -0.3751340000 -1.8964120000
C -1.2871840000 -0.1044490000 -0.0917800000
H -1.3614620000 -0.4233650000 0.9473090000
H -0.6503990000 1.1832010000 0.1273090000
C -2.5780240000 0.2300890000 -0.7525950000
O -0.5896090000 -2.4543050000 -1.2225170000
S 0.6022700000 -3.3557370000 -1.4875550000
O 1.5694620000 -2.6985630000 -2.3839820000
O 0.1718570000 -4.6803470000 -1.9177200000
C 1.4167800000 -3.5038990000 0.0911670000
C 2.7047940000 -4.0358050000 0.1273220000
C 0.7891940000 -3.0722090000 1.2509800000
C 3.3715180000 -4.1089510000 1.3402580000
H 3.1872200000 -4.3667340000 -0.7858360000
C 1.4720770000 -3.1538580000 2.4621570000
H -0.2127590000 -2.6612680000 1.2084890000
C 2.7697980000 -3.6586930000 2.5229630000
H 4.3789940000 -4.5107040000 1.3744690000
H 0.9905390000 -2.8044580000 3.3691330000
C 3.5293840000 -3.6963390000 3.8204220000
H 2.8943640000 -3.4049670000 4.6578190000
H 3.9227850000 -4.6974780000 4.0122810000
H 4.3822610000 -3.0120720000 3.7821060000
O -3.4172120000 -0.9806630000 -0.8108820000
S -4.8888740000 -0.8253190000 -1.4027760000
O -5.4062450000 -2.1742860000 -1.4593970000
O -4.8370540000 -0.0237050000 -2.6097770000
C -5.7452430000 0.0840430000 -0.1489960000
C -6.0361650000 -0.5539410000 1.0559050000
C -6.0886130000 1.4096680000 -0.3741150000
C -6.6900140000 0.1611020000 2.0452620000
H -5.7619530000 -1.5910560000 1.2114890000
C -6.7479150000 2.1092870000 0.6320380000
H -5.8505070000 1.8831260000 -1.3188670000
C -7.0548910000 1.4996650000 1.8483220000
H -6.9261860000 -0.3225640000 2.9870690000
H -7.0258350000 3.1442060000 0.4671570000
C -7.7680920000 2.2517300000 2.9371580000

H -7.1717090000 2.2568140000 3.8528620000
H -7.9625690000 3.2830660000 2.6424690000
H -8.7214530000 1.7712990000 3.1715140000
F -0.2036100000 2.1553980000 0.4212500000
H -3.1361930000 0.9843840000 -0.1907140000
H -2.4254120000 0.5746250000 -1.7768980000
N 3.5843870000 2.3868650000 -0.0294050000
C 3.9628630000 1.6423120000 1.2429170000
C 2.8786010000 1.0685460000 2.2062640000
H 4.5779520000 2.3447180000 1.8091820000
H 4.6118710000 0.8403730000 0.9030340000
O 1.7506540000 0.5317380000 1.5280840000
C 2.4366890000 2.0704170000 3.2766990000
H 1.7878610000 1.5543360000 3.9878120000
H 1.8836910000 2.9193950000 2.8731360000
H 3.3046120000 2.4544310000 3.8172630000
C 3.5549460000 -0.1132520000 2.9027930000
H 3.7878240000 -0.8959860000 2.1776570000
H 2.8795710000 -0.5227920000 3.6570390000
H 4.4795060000 0.2035660000 3.3921030000
H 1.0876150000 1.2212610000 1.3731150000
C 2.9098370000 1.5210310000 -1.0799710000
C 3.5512480000 0.2393600000 -1.6737560000
C 2.6372930000 3.5083680000 0.3554200000
C 1.9827420000 4.4343980000 -0.6955090000
C 2.9219740000 5.1183140000 -1.6795520000
C 1.2594900000 5.4946870000 0.1466440000
O 1.0257980000 3.7355700000 -1.4801490000
C 4.8359140000 0.4799190000 -2.4619770000
O 2.5347250000 -0.0932580000 -2.6273590000
C 3.7344800000 -0.9252000000 -0.7026800000
H 2.7310930000 2.1895000000 -1.9188880000
H 1.9474850000 1.2401630000 -0.6631610000
H 4.7172700000 1.3211820000 -3.1493010000
H 5.0404530000 -0.4174880000 -3.0506200000
H 5.6955590000 0.6615490000 -1.8160730000
H 4.6548380000 -0.8372820000 -0.1239210000
H 3.8072470000 -1.8457730000 -1.2872510000
H 2.8899780000 -1.0143750000 -0.0153410000
H 2.2358370000 -1.0062850000 -2.4850720000
H 3.1895160000 4.1109060000 1.0781370000
H 1.8077220000 3.0277870000 0.8634390000
H 0.4557160000 3.2315100000 -0.8779740000
H 3.7658010000 5.5845520000 -1.1674860000
H 2.3623480000 5.8944640000 -2.2061460000
H 3.2935630000 4.4153070000 -2.4258230000
H 1.9653790000 6.1257100000 0.6914530000
H 0.5833500000 5.0205900000 0.8643390000
H 0.6676250000 6.1215330000 -0.5225060000
C 4.8731520000 2.9589210000 -0.5467920000
H 5.6558030000 2.2145180000 -0.4347950000
H 5.1199240000 3.8361080000 0.0485830000
H 4.7642370000 3.2242100000 -1.5928050000

Electronic Energy (Hartree)	Zero Point Correction (kcal/mol)	Electronic and Zero Point Correction (Hartree)	Gibbs Free Energy (kcal/mol)	Electronic and Gibbs Free Energy (Hartree)	Number of Imaginary Frequencies
−2800.87717	493.42327	−2800.0909	423.1873867	−2800.202778	1

E2_1: post-reaction complex
C -0.2680220000 -0.5936150000 -1.0550010000
H 0.6304410000 -0.8150780000 -0.4894860000
H -0.1586710000 -0.4633800000 -2.1271700000
C -1.4534990000 -0.4999350000 -0.4545550000
H -1.5487950000 -0.6546390000 0.6181860000
H -0.6094060000 1.4206130000 -0.3914570000
C -2.7175910000 -0.1838050000 -1.1857730000
O -0.2465120000 -4.0075000000 -1.4297910000
S 1.2225070000 -3.9936060000 -1.3834640000
O 1.8116740000 -2.8651070000 -2.1503440000
O 1.8527090000 -5.2783640000 -1.7257030000
C 1.6634200000 -3.6842060000 0.3262290000
C 2.9482280000 -3.9987900000 0.7633460000
C 0.7547150000 -3.0877610000 1.1894330000
C 3.3195260000 -3.7053120000 2.0677010000
H 3.6466720000 -4.4757800000 0.0848030000
C 1.1380860000 -2.7965180000 2.4977820000
H -0.2469800000 -2.8658840000 0.8406140000
C 2.4207120000 -3.0995570000 2.9546540000
H 4.3191010000 -3.9538390000 2.4106950000
H 0.4272480000 -2.3315000000 3.1732440000
C 2.8372410000 -2.8074070000 4.3713730000
H 2.0573100000 -2.2668610000 4.9091830000
H 3.0414750000 -3.7373470000 4.9093350000
H 3.7524620000 -2.2100600000 4.3936420000
O -3.6212650000 -1.2931560000 -0.9318120000
S -5.1600410000 -1.0943640000 -1.3462460000
O -5.7500970000 -2.3953440000 -1.1397680000
O -5.2124520000 -0.4670880000 -2.6499700000
C -5.7604740000 0.0386000000 -0.1310140000
C -5.9189210000 -0.4128940000 1.1786690000
C -6.0433700000 1.3482510000 -0.4936930000
C -6.3766890000 0.4772700000 2.1350360000
H -5.6946520000 -1.4407780000 1.4398300000
C -6.5046550000 2.2250500000 0.4829860000
H -5.9122220000 1.6736890000 -1.5184990000
C -6.6762470000 1.8053590000 1.8020440000
H -6.5080460000 0.1417510000 3.1580840000
H -6.7337520000 3.2497880000 0.2131880000
C -7.1820030000 2.7477870000 2.8579400000
H -6.4934900000 2.7798640000 3.7056530000
H -7.2990920000 3.7572380000 2.4636190000
H -8.1500650000 2.4093670000 3.2365890000
F -0.5206820000 2.2957680000 -0.0531330000
H -3.1685000000 0.7366780000 -0.7989760000
H -2.5540110000 -0.0946390000 -2.2598800000
N 3.4606470000 2.5119630000 -0.0966460000
C 3.7196760000 1.9417970000 1.2906410000

C 2.5521750000 1.5149500000 2.2274030000
H 4.3031490000 2.7068570000 1.8066680000
H 4.3738900000 1.0923320000 1.1180970000
O 1.4751400000 0.9104950000 1.5136330000
C 2.0374250000 2.6526510000 3.1113450000
H 1.3241930000 2.2411450000 3.8293370000
H 1.5349340000 3.4422620000 2.5509230000
H 2.8633690000 3.1013620000 3.6669360000
C 3.1397220000 0.4279190000 3.1269070000
H 3.4185650000 -0.4436230000 2.5309500000
H 2.3912630000 0.1268780000 3.8624880000
H 4.0247760000 0.7953340000 3.6529270000
H 0.7528730000 1.5448320000 1.4309660000
C 2.8745250000 1.5171760000 -1.0837050000
C 3.5570540000 0.1765650000 -1.4598500000
C 2.4884580000 3.6658790000 0.0523440000
C 1.9779030000 4.4833890000 -1.1572150000
C 3.0359650000 5.0557480000 -2.0898680000
C 1.1764100000 5.6273930000 -0.5231340000
O 1.1107340000 3.7119190000 -1.9815110000
C 4.9040730000 0.3212700000 -2.1644320000
O 2.6174060000 -0.2737100000 -2.4398430000
C 3.6540120000 -0.8551140000 -0.3373130000
H 2.7641620000 2.0778030000 -2.0092240000
H 1.8850130000 1.2751430000 -0.7083160000
H 4.8452120000 1.0666460000 -2.9614360000
H 5.1487170000 -0.6431530000 -2.6161060000
H 5.7127210000 0.5871280000 -1.4829080000
H 4.5223250000 -0.6879270000 0.3015080000
H 3.7703210000 -1.8400260000 -0.7947020000
H 2.7526920000 -0.8681080000 0.2820710000
H 2.3502170000 -1.1919700000 -2.2507410000
H 2.9587870000 4.3417480000 0.7684950000
H 1.6004610000 3.2362890000 0.5083660000
H 0.3975940000 3.3672310000 -1.4289190000
H 3.8276370000 5.5603000000 -1.5334190000
H 2.5552740000 5.7817430000 -2.7489570000
H 3.4711460000 4.2768420000 -2.7165780000
H 1.8249410000 6.3099900000 0.0302480000
H 0.4158160000 5.2353870000 0.1593450000
H 0.6754200000 6.1813740000 -1.3186730000
C 4.7960250000 3.0144140000 -0.5654660000
H 5.5566040000 2.2894500000 -0.2909060000
H 4.9994240000 3.9597480000 -0.0655150000
H 4.7816650000 3.1431560000 -1.6420610000

Electronic Energy (Hartree)	Zero Point Correction (kcal/mol)	Electronic and Zero Point Correction (Hartree)	Gibbs Free Energy (kcal/mol)	Electronic and Gibbs Free Energy (Hartree)	Number of Imaginary Frequencies
−2800.908723	494.642521	−2800.1205	420.7915555	−2800.238149	0

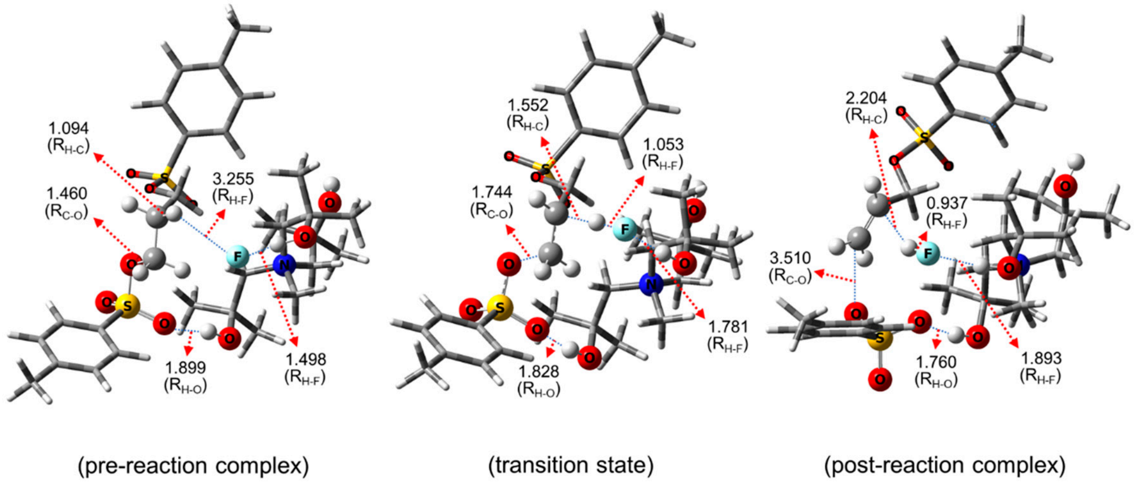

E2_2: pre-reaction complex
C -2.5559990000 1.0945180000 1.2606640000
H -3.5314870000 1.1920630000 1.7437690000
H -2.0347510000 0.1944680000 1.5911530000
C -1.6957650000 2.3080500000 1.5332430000
H -2.1971130000 3.2281930000 1.2232630000
H -1.5629360000 2.3529330000 2.6177170000
C -0.3180880000 2.2284490000 0.9075390000
O -2.7682750000 1.0113030000 -0.1816660000
S -3.5328850000 -0.2657260000 -0.7441910000
O -2.9738530000 -1.4595350000 -0.1290560000
O -3.4855760000 -0.1192220000 -2.1797280000
C -5.1896300000 -0.0662900000 -0.1669970000
C -5.6271850000 -0.7983260000 0.9287830000
C -6.0111820000 0.8570470000 -0.8114700000
C -6.9275920000 -0.6043230000 1.3821250000
H -4.9685040000 -1.5101780000 1.4112520000
C -7.3018720000 1.0349190000 -0.3421700000
H -5.6500860000 1.4143690000 -1.6679730000
C -7.7773250000 0.3097180000 0.7582540000
H -7.2852300000 -1.1732300000 2.2328420000
H -7.9569250000 1.7447790000 -0.8356210000
C -9.1818290000 0.5243070000 1.2481580000
H -9.8993170000 0.3501740000 0.4427150000
H -9.4175720000 -0.1438240000 2.0764830000
H -9.3137180000 1.5557160000 1.5852810000
O -0.4379280000 2.6443960000 -0.4837140000
S 0.7384530000 2.2518560000 -1.4834690000
O 0.3131180000 2.7746330000 -2.7621930000
O 1.0345400000 0.8380390000 -1.3493970000
C 2.1150110000 3.1796690000 -0.8772050000
C 2.1027450000 4.5665630000 -1.0197790000
C 3.1550230000 2.5194460000 -0.2393650000
C 3.1663650000 5.2940970000 -0.5134960000
H 1.2809090000 5.0631060000 -1.5226790000
C 4.2150620000 3.2694940000 0.2613940000
H 3.1386760000 1.4404410000 -0.1405560000
C 4.2352870000 4.6581450000 0.1329320000
H 3.1740890000 6.3735980000 -0.6193330000

H 5.0360070000 2.7658120000 0.7597590000
C 5.3786570000 5.4732150000 0.6694500000
H 6.1160440000 4.8417330000 1.1650990000
H 5.8734400000 6.0154050000 -0.1403910000
H 5.0165530000 6.2152470000 1.3852730000
F -0.1914750000 -0.5680040000 2.1937080000
H 0.3762510000 2.9169000000 1.3951760000
H 0.0563240000 1.2038410000 0.9650460000
N 1.9273360000 -2.4934260000 0.5131610000
C 2.4519640000 -1.1603480000 1.0503480000
H 3.2550770000 -0.8653150000 0.3805530000
H 1.6211580000 -0.4674130000 0.9475100000
C 1.1845830000 -3.2740910000 1.5582900000
H 0.8156040000 -4.1855940000 1.0964330000
H 1.8570940000 -3.5003550000 2.3777550000
H 0.3628460000 -2.6518240000 1.9034050000
C 0.9238780000 -2.1150790000 -0.5698250000
H 1.4722960000 -1.5528040000 -1.3154410000
H 0.2527740000 -1.4175050000 -0.0637920000
C 3.0666880000 -3.3657010000 0.0291640000
H 3.5977650000 -3.6743380000 0.9290560000
H 2.6065370000 -4.2497010000 -0.3978120000
C 0.0052120000 -3.1563910000 -1.2535300000
C 0.6276550000 -4.4519790000 -1.7630930000
H 0.8765220000 -5.1285220000 -0.9439170000
H -0.1112810000 -4.9558030000 -2.3901070000
H 1.5200150000 -4.2656190000 -2.3632440000
C -0.5905940000 -2.3910440000 -2.4426440000
H -1.4032760000 -2.9816750000 -2.8708050000
H -0.9917770000 -1.4245140000 -2.1276840000
H 0.1655860000 -2.2162940000 -3.2119560000
O -1.0405670000 -3.5564810000 -0.3743610000
H -1.6417860000 -2.8068440000 -0.2589390000
C 2.9317200000 -1.0368820000 2.5140800000
C 3.4122810000 0.4214820000 2.6161200000
H 4.2723490000 0.6170150000 1.9687890000
H 2.6032610000 1.1070720000 2.3457120000
H 3.6974600000 0.6188220000 3.6516190000
C 4.0835770000 -1.9549700000 2.9191300000
H 3.7723610000 -2.9986970000 2.9874700000
H 4.9131020000 -1.8812290000 2.2150190000
H 4.4264420000 -1.6535470000 3.9123990000
O 1.8766650000 -1.2396760000 3.4269800000
H 1.0241220000 -0.9232520000 2.9939700000
C 4.1419330000 -2.8517580000 -0.9484810000
C 3.6822460000 -2.0494870000 -2.1652030000
H 3.3186760000 -1.0593740000 -1.8850220000
H 4.5444240000 -1.9098170000 -2.8240450000
H 2.9086540000 -2.5720190000 -2.7311790000
C 4.8721470000 -4.1131520000 -1.4208830000
H 4.2297820000 -4.7208980000 -2.0625250000
H 5.7569760000 -3.8223430000 -1.9927130000
H 5.1933760000 -4.7139090000 -0.5667540000
O 5.0522100000 -2.0571520000 -0.1801420000

H 5.7635460000 -1.7835380000 -0.7719710000

Electronic Energy (Hartree)	Zero Point Correction (kcal/mol)	Electronic and Zero Point Correction (Hartree)	Gibbs Free Energy (kcal/mol)	Electronic and Gibbs Free Energy (Hartree)	Number of Imaginary Frequencies
−2800.91588	497.6696268	−2800.1228	426.6154711	−2800.236025	0

E2_2: transition state
C 1.9193320000 -1.0532120000 1.0328460000
H 2.8620550000 -1.0241110000 1.5757730000
H 1.4536110000 -0.0753510000 0.9393910000
C 1.0638280000 -2.1360750000 1.3427930000
H 1.5342450000 -3.0937420000 1.5525060000
H 0.6952880000 -1.4606590000 2.6902260000
C -0.2713120000 -2.1975200000 0.7107180000
O 2.5552600000 -1.1266110000 -0.5895580000
S 3.4970580000 0.0057400000 -1.0224580000
O 3.0473390000 1.2782020000 -0.4408370000
O 3.6632230000 -0.0317680000 -2.4670580000
C 5.0596480000 -0.3794370000 -0.2682500000
C 5.4801970000 0.3186420000 0.8548590000
C 5.8211770000 -1.4166460000 -0.8040020000
C 6.6921070000 -0.0259310000 1.4477930000
H 4.8753030000 1.1244770000 1.2523820000
C 7.0236890000 -1.7465010000 -0.1998760000
H 5.4814920000 -1.9489690000 -1.6852010000
C 7.4759160000 -1.0581170000 0.9332650000
H 7.0312740000 0.5179990000 2.3224840000
H 7.6267480000 -2.5491530000 -0.6112310000
C 8.7854940000 -1.4350030000 1.5694060000
H 9.6030080000 -1.3399060000 0.8503630000
H 9.0040580000 -0.8005030000 2.4285670000
H 8.7640610000 -2.4755610000 1.9031250000
O -0.1987200000 -2.7306450000 -0.6864790000
S -1.2987720000 -2.2243570000 -1.7079690000
O -1.0649670000 -2.9726580000 -2.9242500000
O -1.2815580000 -0.7711090000 -1.7600050000
C -2.8272550000 -2.7339110000 -0.9717190000
C -3.1415600000 -4.0910990000 -0.9656400000
C -3.6542380000 -1.7930910000 -0.3710670000
C -4.3139780000 -4.5014400000 -0.3506690000
H -2.4828680000 -4.8112780000 -1.4374770000
C -4.8267480000 -2.2252000000 0.2395190000
H -3.3991410000 -0.7395930000 -0.3843100000
C -5.1718990000 -3.5775110000 0.2587300000
H -4.5707420000 -5.5551070000 -0.3396010000
H -5.4812360000 -1.4989100000 0.7097190000
C -6.4517800000 -4.0391170000 0.8975000000
H -6.8369510000 -3.2923590000 1.5924400000
H -7.2129850000 -4.2129510000 0.1312740000
H -6.3033190000 -4.9786360000 1.4327600000
F 0.4021660000 -0.9260030000 3.5484920000
H -0.9555500000 -2.8713200000 1.2305140000
H -0.7184730000 -1.2056410000 0.6392350000

N -1.1546710000 2.5820770000 0.4926900000
C -1.4952370000 1.3036100000 1.2364230000
H -2.1925220000 0.7722100000 0.5934220000
H -0.5600430000 0.7454800000 1.2765830000
C -0.2767880000 3.4484840000 1.3424310000
H -0.0414530000 4.3537520000 0.7912260000
H -0.7917430000 3.6806410000 2.2680800000
H 0.6353190000 2.8929640000 1.5526370000
C -0.3670830000 2.1468950000 -0.7362520000
H -1.0471610000 1.5661350000 -1.3464330000
H 0.3878840000 1.4563240000 -0.3551990000
C -2.4072870000 3.3629440000 0.1408050000
H -2.7799350000 3.7547570000 1.0841850000
H -2.0759880000 4.2076500000 -0.4530730000
C 0.4099020000 3.1623230000 -1.6110890000
C -0.3075150000 4.4326800000 -2.0547570000
H -0.4483200000 5.1307120000 -1.2279990000
H 0.3249350000 4.9280550000 -2.7946030000
H -1.2729830000 4.2196280000 -2.5160620000
C 0.7984290000 2.3534610000 -2.8564850000
H 1.4901350000 2.9478470000 -3.4568150000
H 1.2964560000 1.4196400000 -2.5829730000
H -0.0814480000 2.1152040000 -3.4594400000
O 1.5828850000 3.6038940000 -0.9413360000
H 2.1514610000 2.8306670000 -0.7991060000
C -1.9940470000 1.3242350000 2.7072010000
C -2.5983130000 -0.0703060000 2.9158160000
H -3.5155440000 -0.1794210000 2.3321280000
H -1.8925060000 -0.8515820000 2.6243750000
H -2.8299110000 -0.1990940000 3.9753780000
C -3.0230260000 2.3663770000 3.1374270000
H -2.6076650000 3.3746120000 3.1625090000
H -3.9003200000 2.3495980000 2.4942080000
H -3.3231100000 2.1183160000 4.1580780000
O -0.8773330000 1.5059860000 3.5715640000
H -0.3605340000 0.6833480000 3.5636660000
C -3.6034360000 2.7117580000 -0.5854930000
C -3.3290870000 2.0124900000 -1.9167160000
H -2.8442200000 1.0456720000 -1.7757500000
H -4.2883260000 1.8309150000 -2.4103980000
H -2.7197240000 2.6267470000 -2.5828980000
C -4.5734580000 3.8754080000 -0.8201650000
H -4.1644670000 4.5856350000 -1.5425360000
H -5.5117390000 3.4824690000 -1.2197860000
H -4.7856580000 4.3989650000 0.1148670000
O -4.2106340000 1.7765520000 0.3138670000
H -5.0281160000 1.4709220000 -0.0990020000

Electronic Energy (Hartree)	Zero Point Correction (kcal/mol)	Electronic and Zero Point Correction (Hartree)	Gibbs Free Energy (kcal/mol)	Electronic and Gibbs Free Energy (Hartree)	Number of Imaginary Frequencies
−2800.874335	493.9692033	−2800.0871	422.8610818	−2800.200463	1

E2_2: post-reaction complex

C 2.2282980000 -2.2536520000 -0.9017040000
H 3.0447980000 -2.9219640000 -0.6441400000
H 2.4841590000 -1.2916150000 -1.3368680000
C 0.9572990000 -2.6057830000 -0.7189720000
H 0.6912670000 -3.5788960000 -0.3146920000
H 1.6681050000 -1.7766240000 1.1953000000
C -0.1749090000 -1.6929650000 -1.0608060000
O 3.8403580000 0.5165050000 -2.3328900000
S 4.1304250000 1.0086480000 -0.9752650000
O 2.8958370000 1.3959680000 -0.2356340000
O 5.1641670000 2.0531250000 -0.9285470000
C 4.7673940000 -0.4044500000 -0.0782750000
C 4.3613060000 -0.6471310000 1.2259800000
C 5.6607430000 -1.2628190000 -0.7149590000
C 4.8482180000 -1.7665710000 1.8972510000
H 3.6480280000 0.0158800000 1.6998960000
C 6.1458210000 -2.3694990000 -0.0322890000
H 5.9538650000 -1.0779140000 -1.7423430000
C 5.7481570000 -2.6378830000 1.2833190000
H 4.5173880000 -1.9671290000 2.9109400000
H 6.8374470000 -3.0439690000 -0.5269580000
C 6.2942690000 -3.8354450000 2.0130750000
H 7.3344040000 -3.6630920000 2.3044360000
H 5.7199080000 -4.0411420000 2.9171280000
H 6.2759990000 -4.7228830000 1.3768950000
O -0.8675760000 -2.2956330000 -2.1896530000
S -2.2844990000 -1.6513790000 -2.5774860000
O -2.6291870000 -2.2707390000 -3.8344980000
O -2.1729390000 -0.2084580000 -2.4829880000
C -3.3534850000 -2.2369220000 -1.2981910000
C -3.6227690000 -3.6039110000 -1.2313740000
C -3.8919290000 -1.3398460000 -0.3873820000
C -4.4543030000 -4.0659660000 -0.2260390000
H -3.1928650000 -4.2898730000 -1.9522860000
C -4.7316940000 -1.8255230000 0.6107240000
H -3.6675620000 -0.2819350000 -0.4513920000
C -5.0213420000 -3.1861200000 0.7068180000
H -4.6732730000 -5.1261880000 -0.1593300000
H -5.1611860000 -1.1341600000 1.3279770000
C -5.9261400000 -3.7160490000 1.7833080000
H -6.2521470000 -2.9203530000 2.4530810000
H -6.8102620000 -4.1828910000 1.3415510000
H -5.4133330000 -4.4808070000 2.3716550000
F 1.5307370000 -1.6142340000 2.1073520000
H -0.8765560000 -1.6097080000 -0.2241960000
H 0.1758180000 -0.7016440000 -1.3544360000
N -0.7030200000 2.3435980000 0.9245420000
C -0.9671140000 0.8968770000 1.3083440000
H -1.8310030000 0.5868340000 0.7217770000
H -0.0923460000 0.3458600000 0.9542470000
C 0.3577320000 2.9282290000 1.8099520000
H 0.5161480000 3.9606510000 1.5141230000
H 0.0384980000 2.8604340000 2.8435370000
H 1.2711340000 2.3539150000 1.6609920000

C -0.1640730000 2.2951130000 -0.5014770000
H -0.9780570000 1.9431660000 -1.1278990000
H 0.6028230000 1.5199540000 -0.4750130000
C -1.9426830000 3.2087290000 1.0671560000
H -2.0963720000 3.3163810000 2.1385700000
H -1.6629350000 4.1820410000 0.6770000000
C 0.5221920000 3.5176420000 -1.1662110000
C -0.2631440000 4.8224050000 -1.2322670000
H -0.3149190000 5.3047990000 -0.2547990000
H 0.2703300000 5.5019800000 -1.9007930000
H -1.2713560000 4.6787740000 -1.6221120000
C 0.8167490000 3.0201060000 -2.5897750000
H 1.3843430000 3.7902390000 -3.1155910000
H 1.4236750000 2.1100650000 -2.5541010000
H -0.1021920000 2.8154170000 -3.1454940000
O 1.7444550000 3.8464500000 -0.5275130000
H 2.2665330000 3.0267670000 -0.4384310000
C -1.1282200000 0.4794020000 2.7930000000
C -1.6552230000 -0.9584930000 2.7278250000
H -2.6785620000 -0.9779950000 2.3473100000
H -1.0269370000 -1.5785690000 2.0821520000
H -1.6410240000 -1.3840200000 3.7332570000
C -2.0470860000 1.3000510000 3.6919230000
H -1.6410580000 2.2897400000 3.9049930000
H -3.0363360000 1.4049010000 3.2518200000
H -2.1290490000 0.7718540000 4.6445640000
O 0.1448100000 0.4935960000 3.4357070000
H 0.6994020000 -0.1710680000 3.0081630000
C -3.3059440000 2.8302690000 0.4538760000
C -3.3537470000 2.5495890000 -1.0453740000
H -2.8799150000 1.6029470000 -1.3012760000
H -4.4041660000 2.4817830000 -1.3444400000
H -2.8920770000 3.3504130000 -1.6247910000
C -4.2021250000 4.0336570000 0.7683650000
H -3.8731000000 4.9195690000 0.2205380000
H -5.2268310000 3.8047300000 0.4643370000
H -4.1974220000 4.2528370000 1.8384830000
O -3.8045170000 1.6854550000 1.1559110000
H -4.7282640000 1.5707150000 0.8986120000

Electronic Energy (Hartree)	Zero Point Correction (kcal/mol)	Electronic and Zero Point Correction (Hartree)	Gibbs Free Energy (kcal/mol)	Electronic and Gibbs Free Energy (Hartree)	Number of Imaginary Frequencies
−2800.918295	494.4072049	−2800.1304	420.0272489	−2800.24894	0
